# Editorial: Community series in translational insights into mechanisms and therapy of organ dysfunction in sepsis and trauma, volume II

**DOI:** 10.3389/fimmu.2023.1215888

**Published:** 2023-05-25

**Authors:** Borna Relja, Sina Maren Coldewey, Pietro Ghezzi, Lukas Martin, Christoph Thiemermann

**Affiliations:** ^1^ Department of Trauma, Hand, Plastic and Reconstructive Surgery, Translational and Experimental Trauma Research, University Hospital Ulm, Ulm University, Ulm, Germany; ^2^ University Hospital Jena, Department of Anesthesiology and Intensive Care Medicine, Jena University Hospital, Jena, Germany; ^3^ University of Urbino Carlo Bo, Department of Biomolecular Sciences, Urbino, Italy; ^4^ University Hospital RWTH Aachen, Department of Intensive Care and Intermediate Care, Aachen, Germany; ^5^ Queen Mary University of London, William Harvey Research Institute, Barts and the London School of Medicine and Dentistry, Centre for Translational Medicine and Therapeutics, London, United Kingdom

**Keywords:** sepsis, trauma, infections, complications, therapy, outcome

Critically ill patients with sepsis often experience life-threatening organ failure caused by a dysregulated response to inflammation. While there is a good understanding of some of the key signaling pathways involved in sepsis-related inflammation, the development of effective organ-protective therapeutic strategies is still limited. At present, therapeutic approaches for sepsis primarily involve source control, antibiotics, supportive care, and early goal-directed therapy. However, there is a lack of specific and effective treatments for the inflammatory response associated with sepsis, which can lead to multiple organ dysfunction in clinical patients. In addition, individuals who survive the initial acute stage of sepsis may develop an immuno-suppressive state, leaving them at an increased risk of detrimental secondary infections and high mortality rates. Therefore, relying solely on anti-inflammatory therapy may not be enough to successfully treat sepsis.

In their original article, Mohammad et al. discuss how Aquaporins, specifically RG100204, a new Aquaporin-9 inhibitor, can regulate crucial mechanisms in a cecal ligation and puncture (CLP) induced model of polymicrobial infection (sepsis) by reducing cardiac dysfunction (systolic and diastolic), renal dysfunction, and hepatocellular injury. One notable finding is that administering RG100204 orally, even 3 hours after the onset of polymicrobial sepsis, can reduce the activation of the NLRP3 inflammasome pathway and myeloperoxidase activity in the lungs, thereby reducing cardiac and renal dysfunction caused by severe sepsis. This and previous reports suggest that AQP9 could be a promising drug target in the treatment of polymicrobial sepsis, and warrant further analysis in future trials.

Zhang et al. have shown that Erythropoietin (EPO), a glycoprotein that is regulated by hypoxia-inducible factor 1α (HIF-1α), is naturally induced in endotoxin-tolerant macrophages upon initial exposure to LPS. This study is the first to demonstrate that EPO plays a role in regulating the functional re-programming of endotoxin-tolerant macrophages.

When endotoxin-tolerized macrophages were exposed to EPO, they expressed fewer proinflammatory genes, such as *Il1b*, *Il6*, and *Tnfa*, and more host-protective genes, such as *Cnlp*, *Marco*, and *Vegfc*. This effect was achieved through the upregulation of *Irak3* and *Wdr5 via* the PI3K/AKT pathway upon secondary exposure to LPS. The authors also found that LPS-tolerized mice treated with EPO were protected against secondary infection with *E. coli* and had improved outcomes after sepsis. While more research is needed to translate these findings to clinical settings, this drug presents new opportunities for the treatment of sepsis.

Dennhardt et al. discovered that EPO and its non-hematopoietic analog, pyroglutamate helix B surface peptide (pHBSP), can provide tissue protection through the innate repair receptor (IRR), independent of their hematopoietic properties *via* the EPO receptor (EPO-R) homodimer. Their research also showed that EPO signaling plays a role in the pathology of Hemolytic-uremic syndrome (HUS) caused by infections with Shiga toxin (Stx)-producing *E. coli*, as evidenced by elevated levels of endogenous EPO in patients with HUS, piglets, and mice subjected to preclinical HUS models. Moreover, the protective effects of pHBSP and EPO were linked to decreased renal oxidative stress, and pHBSP was associated with reduced nitrosative stress and less KIM-1 expression in Stx-challenged mice, without any thromboembolic complications or other adverse side effects. The authors provide evidence that treating mice with HUS with EPO or pHBSP improves 7-day survival and disease outcome, and suggest that targeting the EPO-R/IRR axis could be a promising approach for treating patients with hemolytic anemia in HUS in future clinical trials.

Prior research has demonstrated that inhibiting Bruton’s tyrosine kinase (BTK), a key factor in the recruitment and function of immune cells, can enhance renal function in experimental cases of sepsis and lupus nephritis. In a new study by Kröller et al., it was found that two FDA-approved BTK inhibitors, ibrutinib and acalabrutinib, can limit the progression of HUS in mice by reducing the activation of phospholipase-C-gamma-2 in the spleen, thereby decreasing the invasion of BTK-positive cells like neutrophils into the kidneys. Treatment with ibrutinib resulted in a decline in the infiltration of macrophages, improvement in acute kidney injury and dysfunction markers (NGAL and urea), and a decrease in hemolysis (bilirubin and LDH activity). These findings suggest that inhibiting BTK could be a promising and effective therapeutic approach for HUS by reducing the infiltration of immune cells in the kidneys, as demonstrated in a murine model.

Numerous clinical and experimental studies have indicated that the glycocalyx, which is involved in endothelial dysfunction that leads to sepsis-induced multiple organ failure, could be a promising early target for endothelial injury during infection. Urban et al. conducted experiments on mice with CLP-induced sepsis and administered Colivelin, a synthetic derivative of the mitochondrial peptide humanin. The results showed that Colivelin restored endothelial stability and reduced the infiltration of inflammatory cells in the lungs, kidneys, and liver, along with decreasing the systemic release of pro-inflammatory cytokines. These effects were associated with the inhibition of the signal transducer and activator of transcription 3 and the activation of the AMP-activated protein kinase in the aorta and lungs. When used in conjunction with standard fluid resuscitation and antibiotics, Colivelin improved the long-term recovery and health outcomes of septic mice.

Alves et al. evoked polymicrobial sepsis in WT mice and knockout mice for ICOS, ICOSL and OPN genes. Mice that received a soluble recombinant form of ICOS ICOS-Fc exhibited a decrease in plasma cytokine levels (TNF-α, IL-1β, IL-6, IFN-γ, and IL-10), liver injury (AST and ALT), kidney dysfunction (creatinine and urea), and local activation of FAK, P38 MAPK, and NLRP3 inflammasome caused by sepsis. The authors presented evidence that ICOS-Fc works on both sides of the ICOS-ICOSL interaction, as the protective effect was absent in septic knockout mice for the ICOS or ICOSL genes, while it was preserved in OPN knockout mice. These findings suggest that pharmacologically modifying the ICOS-ICOSL pathway may have beneficial effects and that there may be a potential cross-talk mechanism involving the FAK-p38-NLRP3 inflammasome axis in counteracting sepsis-induced inflammation and organ dysfunction.

Zheng et al. investigated the potential use of immune-related genes (IRGs) as biomarkers for developing diagnostic and prognostic models for sepsis outcomes. Their study aimed to understand the immune microenvironment of circulating immune cells, assess the immunosuppression state in sepsis, and develop a prognostic model based on IRGs to identify patients at high risk and predict 28-day mortality. The authors demonstrated that the immune response is critical in the development of sepsis. Using a Cox prediction model, they identified 22 differentially expressed immune-related genes (DEIRGs) that classified patients into low-risk and high-risk groups and constructed the prognostic model. The regulatory network between transcription factors (TFs) and prognostic DEIRGs provided novel molecular mechanisms in sepsis. The prognostic model showed high accuracy and performance in identifying patients at high risk and predicting 28-day mortality in sepsis patients.

Wang et al. conducted a study to evaluate the therapeutic effects of Human Umbilical Cord Mesenchymal Stem Cells (HUMSCs) intervention on lung tissue in juvenile septic rats. For the first time, they performed proteomic and phosphorylated proteomic screening and analysis on the lung tissue to identify differentially expressed proteins and significantly changed phosphorylation sites after 24 hours of HUMSCs intervention. The results revealed that 213 proteins and 971 phosphorylation sites showed significant changes in the therapy group. The authors found that Tenascin-C could be the key protein responsible for promoting lung injury repair in juvenile septic rats through HUMSCs intervention. Furthermore, the study suggested that HUMSCs may activate Tenascin-C-mediated PGE2 release and improve endothelial cell functional barrier, leading to the recovery of gas-blood barrier function in lung tissue. Phosphorylation analysis revealed that HUMSCs may regulate the phosphorylation of VEGFA through the EGFR tight junction pathway, thus alleviating inflammatory injury and improving the permeability of the endothelial barrier in lung tissue. Overall, the study identified potential new therapeutic targets for HUMSCs to alleviate lung injury in a juvenile sepsis rat model.

Martinez-Orengo et al. have proposed a new approach for evaluating and measuring organ-level immunoreactivity and associated dysfunction in systemic inflammatory response syndrome (SIRS) and sepsis. Using a PET imaging tracer [^18^F]DPA-714 for the translocator protein (TSPO), the authors were able to detect increased binding in the brain, lungs, liver, and bone marrow in a rat model of SIRS induced by LPS administration. The *in vivo* PET/CT scans were validated by *in vitro* measures of TSPO expression and immunofluorescent staining. The study revealed brain, liver, and lung inflammation, spleen monocytic efflux/lymphocytic activation, and suggested increased bone marrow hematopoiesis. The authors suggest that [^18^F]DPA-714 PET could be used as a noninvasive tool to monitor organ-level inflammation in different organs (lung, liver, bone marrow, brain, and spleen) in SIRS and sepsis and to evaluate therapeutic interventions aimed at decreasing or controlling inflammation.

Amoafo et al. have shown that purinergic signaling, a newly identified regulatory mechanism in immune cell physiology, may be regulated differently in male and female mice in response to hormones. They found that deficiency in the P2Y_12_ receptor but not the P2Y_1_ receptor decreased activity of MPO in lungs and kidneys, platelet-leukocyte interaction, and platelet activation in male mice with sepsis, but not in female mice. Treatment with selective antagonists for P2Y_12_ or P2Y_1_ receptors also decreased sepsis-induced MPO levels, aggregate formation, and platelet activation in male mice, but not in female mice. These findings were supported by *in vitro* experiments with human T lymphocytes, where blocking P2Y_1_ or P2Y_12_ receptors had a sex-dependent effect on cell growth and secretion. These results suggest that drug targeting purinergic signaling in sepsis should be carefully tailored to the sex of the patient in future targeted therapies.

The focus of this Research Topic is to shed new light on the molecular mechanisms that contribute to inflammation during septic conditions, with particular attention given to preclinical and translational studies aimed at developing new therapeutic strategies to protect organs.

## Author contributions

All authors listed have made a substantial, direct, and intellectual contribution to the work and approved it for publication.

